# Analytical Interference by Contrast Agents in Biochemical Assays

**DOI:** 10.1155/2017/1323802

**Published:** 2017-04-10

**Authors:** Sigrid Otnes, Niels Fogh-Andersen, Janne Rømsing, Henrik S. Thomsen

**Affiliations:** ^1^Faculty of Health and Medical Sciences, University of Copenhagen, Copenhagen, Denmark; ^2^Department of Clinical Biochemistry, Copenhagen University Hospital Herlev, Herlev, Denmark; ^3^Department of Radiology, Copenhagen University Hospital Herlev, Herlev, Denmark

## Abstract

**Objective:**

To provide a clinically relevant overview of the analytical interference by contrast agents (CA) in laboratory blood test measurements.

**Materials and Methods:**

The effects of five CAs, gadobutrol, gadoterate meglumine, gadoxetate disodium, iodixanol, and iomeprol, were studied on the 29 most frequently performed biochemical assays. One-day-old plasma, serum, and whole blood were spiked with doses of each agent such that the gadolinium agents and the iodine agents reached concentrations of 0.5 mM and 12 mg iodine/mL, respectively. Subsequently, 12 assays were reexamined using 1/2 and 1/4 of these CA concentrations. The results were assessed statistically by a paired Student's *t*-test.

**Results:**

Iodixanol produced a negative interference on the bicarbonate (*p* = 0.011), lactate dehydrogenase (*p* < 0.0001), and zinc (*p* = 0.0034) assays and a positive interference on the albumin (*p* = 0.0062), calcium (*p* < 0.0001), ionized calcium (*p* = 0.0086), iron (*p* < 0.0001), and potassium (*p* = 0.0003) assays. Iomeprol produced a negative interference on the bicarbonate (*p* = 0.0057) and magnesium (*p* = 0.0001) assays and a positive interference on the calcium (*p* < 0.0001) and potassium (*p* = 0.0012) assays. Gadoxetate disodium produced a negative interference on the iron (*p* < 0.0001) and zinc (*p* < 0.0001) assays and a positive interference on the sodium (*p* = 0.032) assay.

**Conclusion:**

CAs cause analytical interference. Attention should be given to the above-mentioned analyte-CA combinations when assessing laboratory blood test results obtained after CA administration.

## 1. Introduction

Radiological imaging and laboratory blood tests are used together to diagnose and treat patients. To optimize the physician's evaluation and reduce the inconvenience for the patient, ideally, all planned examinations and tests should be performed on one day or within a few days. Due to the patients' busy schedules, blood samples may be taken after a computed tomography (CT) or a magnetic resonance (MR) examination. To ensure patient safety, results obtained using patient blood samples must be valid.

The gadolinium-based contrast agents (GdCA) and the iodine-based contrast agents (ICA) are the contrast agents (CA) that in their class are among the most frequently used agents [1]. GdCAs have been reported to interfere with the determination of calcium [2–13], iron [5, 12, 14, 15], zinc, copper [5, 11, 16], angiotensin-converting enzyme, creatinine, bilirubin, protein [5, 12, 13, 17–21] and magnesium [12], and the ICAs interfere with the copper, iron, phosphate, bilirubin, and protein assays [5, 19, 22–27]. The effects of CAs, especially ICAs, on laboratory blood tests have not been studied systematically; therefore, guidelines are based on CA elimination instead of assay-specific recommendations [23, 25, 28, 29]. We performed an in vitro study on the analytical interference caused by contrast agents in laboratory blood tests.

A study was conducted to test the effect of one agent from each of the most frequently used classes of CAs on the most frequently performed laboratory blood tests.

## 2. Materials and Methods

### 2.1. Contrast Agents

The analytical experiments were performed at the Department of Clinical Biochemistry, Copenhagen University Hospital Herlev between February 2 and March 17, 2015. The following five commercially available CAs were selected based on their clinical relevance, that is, among the most commonly used agents in their class world-wide. In addition, they represent different properties of GdCAs and ICAs: a nonionic monomer, low-osmolar ICA, and iomeprol (Iomeron, Bracco Imaging SpA, Milano, Italy); a nonionic dimer, iso-osmolar ICA, and iodixanol (Visipaque, GE Healthcare, Little Chalfont, United Kingdom); an ionic linear GdCA and gadoxetate disodium (Primovist, Bayer Healthcare, Berlin, Germany); an ionic macrocyclic GdCA and gadoterate meglumine (Dotarem, Guerbet, Villepinte, France); and a nonionic macrocyclic GdCA, gadobutrol (Gadovist, Bayer Healthcare, Berlin, Germany).

### 2.2. Preparation of Solutions

Test solutions were prepared by serial dilutions of the various CAs with saline (0.9%), from which 40 *μ*L was added to 1 mL of pooled plasma and pooled serum, and 48 *μ*L was added to 1.20 mL of whole blood. This procedure was repeated 14 times with the initial high concentration of the CAs, providing results from 14 different pools. The haematology assays were repeated 14 times on 14 samples of whole blood. The final concentration of 0.5 mM gadolinium (Gd) and 12 mg iodine/mL corresponds to the estimated peak serum concentration achieved in vivo at a standard dose [15, 30]. The standard dose is defined as 0.1 mmol/kg GdCA and 120 mL of a 300 mg iodine/mL ICA [28]. The control samples were corrected for the dilution using 0.9% saline. The samples were collected from lithium heparin-treated blood in a gel separator tube (Vacuette® LH Lithium Heparin Sep, Greiner Bio-One); citrate/citric acid buffer-treated blood (Vacuette FC Mix, Greiner Bio-One); clot activator-treated blood in a gel separator tube (Vacuette Z Serum Sep Clot Activator, Greiner Bio-One); clot activator-treated blood (Vacuette Z Serum Clot Activator, Greiner Bio-One); citrated plasma (Vacuette 9NC Coagulation Sodium Citrate, Greiner Bio-One); and K3 EDTA-treated blood (Vacuette K3E K3EDTA, Greiner Bio-One); and analytes were tested from the most appropriate tube (Table 1), from which all patient demographic data were removed [31].

### 2.3. Analytes and Biochemical Assays

The analysers and biochemical assays that were used are listed in Table 1. Initially, the 29 biochemical assays were tested on 14 test solutions, one measurement for each prepared test solution, and 12 assays were retested with lower CA concentrations based on the first results. The test solutions were incubated at room temperature and then analysed after 2–4 h and again after 24 h. All reagents and assays were obtained from the manufacturer of the analyser and used according to the manufacturers' instructions.

The assays that were performed on the Vitros® 5.1 Chemistry System are dry chemistry methods that use MicroSlides that contain the necessary reagents in dried form. There are two types of MicroSlides; the colorimetric/rate (CM/Rate) and the immunorate (IR) slides measure the analyte activity or concentration, whereas the potentiometric (PM) slides measure potassium and sodium by direct potentiometry [32].

The three assays performed in the KonelabTM PRIME 60i Clinical Chemistry Analyzer use wet chemistry methods [33]. The two analytes C-reactive proteins (CRP) and zinc are measured by turbidimetric and colorimetric principles, respectively. Ionized calcium is measured by direct potentiometry using saline with calcium chloride as the calibrator. The results are reported as concentrations after adjusting the results to a plasma pH of 7.4 [33].

The coagulation factors II + VII + X assay that was performed on an ACL TOP® 700 CTS is a wet chemistry method that is based on turbidimetric principles and uses Owren's prothrombin time (PT) reagents (Medirox AB, Nyköping, Sweden).

The haematology analytes were assayed on the ADVIA® 2120i System with Autoslide using wet chemistry, flow cytometry, and peroxidase staining methodologies, with the exception of the haemoglobin assay that uses a standard cyanmethemoglobin colorimetric method [34].

None of the reagent manufacturers identified contrast agents as interferents or noninterferents in their assay kit inserts. Iodide was specified as a noninterferent in the glucose and creatinine assays, which were performed on the Vitros 5.1 Chemistry System.

The assays that exhibited a clinically relevant change, i.e., exceeding the Desirable Analytical Quality Specifications for bias, in the presence of a CA were repeated at reduced concentrations, 6 and 3 mg iodine/mL and 0.25 and 0.125 mM Gd. The assays were repeated six times on six different pools.

### 2.4. Statistical Analysis

A paired Student's *t*-test was used to assess the statistical significance of the differences observed. The ANOVA test was used to analyse the two parameter concentrations and the type of drug when relevant. The statistical tests were performed using the statistical computer software R 3.1.2 for Windows (an open source software initially written by Robert Gentleman and Ross Ihaka of the Statistics Department of the University of Auckland) [35]. Desirable Analytical Quality Specifications for imprecision, bias, and total error derived from intra- and interindividual biologic variation were used to assess the clinical relevance of the statistically significant changes in the analyte concentrations [36]. The results are shown as percentage change as follows: Change% = ((*C*_CA_ − *C*_Control_)/*C*_control_)*∗*100, in which a minus or a plus sign indicates a decrease or increase in the results, respectively.

## 3. Results

The results of the assays that exhibited clinically relevant interference by a contrast agent concentration of 0.5 mM Gd and 12 mg iodine/mL are presented in Table 2. Only the results that were obtained after 2–4 h of incubation are presented. Interference by iodixanol, iomeprol, and gadoxetate disodium was observed in a total of 10 assays performed on the Vitros 5.1 FS Chemistry System and KonelabTM PRIME 60i Clinical Chemistry Analyzer.

For some of the assays, the incubation time was a factor, as clinically relevant changes were observed after 24 h of incubation but not after 2–4 h of incubation. The assays that were affected by the incubation time were the albumin, urea, creatinine, ionized calcium, coagulation factors, and haematology assays.

**Figure 1:**
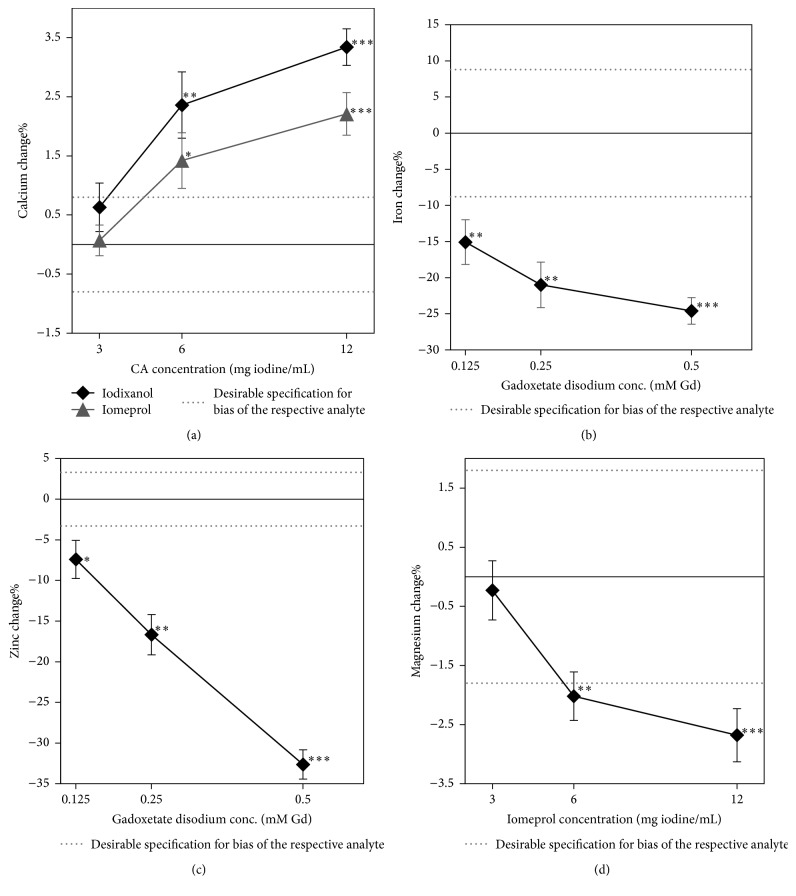
Interferogram presenting iodixanol and iomeprol interference in calcium assays (a), gadoxetate disodium interference in the iron (b) and zinc assays (c), and iomeprol interference in the magnesium assay (d). The error bars represent the SEM.^*∗*^*p* < 0.05 from Student's *t*-test, ^*∗∗*^*p* < 0.01 from Student's *t*-test, and ^*∗∗∗*^*p* < 0.001 from Student's *t*-test.

Some assays did not show a clinically relevant change at the high dose but rather at 1/2 or 1/4 of this dose. Those assays and their respective CAs are the following: bilirubin, iodixanol (6 mg iodine/mL) and iomeprol (6 mg iodine/mL); calcium, gadoxetate disodium (0.125 mM Gd); coagulation factors, iomeprol (6 mg iodine/mL); and magnesium, iodixanol (6 mg iodine/mL). The assays that exhibited clinically relevant changes at high and reduced concentrations were serum calcium, iron, sodium, zinc, and magnesium and are shown in Figure 1.

### 3.1. Colorimetric Calcium Assay

A positive interference was observed with both ICAs in the colorimetric calcium assay using Arsenazo III dye (Figure 1(a)). This effect is concentration dependent for both agents, iodixanol (*p* = 0.0005) and iomeprol (*p* = 0.003). The effect with iodixanol was significantly different when compared with iomeprol (*p* = 0.0053), according to the ANOVA test.

### 3.2. Colorimetric Iron Assay

A negative interference was observed with gadoxetate disodium in the iron assay using a 3-pyridine sulphonamide dye (Figure 1(b)). The figure indicates an increase in the effect with increasing GdCA concentrations, but this is not statistically significant (*p* = 0.063), according to the ANOVA test.

### 3.3. Colorimetric Zinc Assay

A negative interference was observed with gadoxetate disodium in the zinc assay using a 5-Br-PAPS dye (Figure 1(c)). This effect is concentration dependent (*p* < 0.0001), according to the ANOVA test.

### 3.4. Colorimetric Magnesium Assay

A negative interference was observed with iomeprol in the magnesium assay using a formazan dye derivative (Figure 1(d)). The effect is concentration dependent (*p* = 0.0056), according to the ANOVA test.

### 3.5. Potentiometric Sodium Assay

A negative interference with both ICAs was observed in the sodium assay, which is a direct (undiluted) potentiometric assay using methyl monensin as an ionophore (Table 2). The effect is clinically relevant at reduced concentrations and is concentration dependent for iomeprol (*p* = 0.022). The effect with iodixanol was significantly different from that of iomeprol (*p* = 0.038), according to the ANOVA test. Unfortunately, the results of this assay are biased by the use of saline when correcting for dilution.

No analytical interference was observed in any of the 29 assays with the two nonspecific extracellular GdCAs, gadobutrol and gadoterate meglumine, and no clinically relevant effects were observed with either CAs on the alanine amino transferase, alkaline phosphatase, aspartate amino transferase, glucose, and urea assays using the Vitros 5.1 FS Chemistry System and the corresponding dry chemistry method. Furthermore, the CAs did not induce analytical interference in the C-reactive protein assay that was performed on the KonelabTM PRIME 60i Clinical Chemistry Analyzer, or the haematology assays performed on the ADVIA 2120i System.

## 4. Discussion

The main finding in this study is the extent of analytical interference observed with ICAs, some of which were previously unreported. Interestingly, a larger effect is produced by iodixanol than by iomeprol. The analytes at risk of analytical interference by CAs are mainly endogenous ions. The analytical interferences that were observed only at the high concentrations, 12 mg iodine/mL and 0.5 mM Gd, are considered clinically relevant when performed shortly after injections of CAs or for patients with impaired renal function.

The positive interference by nonionic ICAs in assays for calcium has not been reported previously. In 1994, Hayakawa et al. [27] reported a negative interference by ionic ICAs in the determination of ionized calcium, and the effect was attributed to the unbound anions of the ionic ICAs and their potentiation of calcium binding. This mechanism of action cannot possibly explain the observed effect in our study, which is a positive interference by nonionic ICAs. ICAs formulated with calcium binding additives have been known to cause spurious hypocalcemia from interference [37, 38]. The high stability of iomeprol enables it to be formulated without a calcium chelating agent, whereas iodixanol is formulated with sodium calcium ethylenediaminetetraacetic acid (Na2-Ca-EDTA), a chelate that does not bind additional calcium [27, 39, 40]. Iodixanol is formulated isotonically with blood by the addition of sodium and calcium electrolytes, which may explain the larger effect in the colorimetric assay compared to iomeprol. A mechanism of action based on the interaction between ICA and the chromophore may be proposed and is justified by the lack of interference from iomeprol on the ionized calcium assay. In vivo studies have not demonstrated this effect by iodixanol in blood calcium measurements, thus indicating an artefactual in vitro effect [39, 41].

The lack of an effect of gadoxetate disodium, gadoterate meglumine, and gadobutrol on the calcium assays is supported by others, as analytical interference in calcium assays has been attributed to the reduced complex stability and excess ligand in the formulation of the nonionic linear GdCAs [8, 9, 11, 15]. Evidence points to the fact that gadopentetate dimeglumine, gadoterate, gadoteridol, gadobutrol, gadobenate dimeglumine, and gadoxetate disodium do not cause analytical interference in colorimetric calcium assays, regardless of which colorimetric technique was used [2, 6–9, 11–13]. Only nonionic linear GdCAs are relevant when considering Gd chelate binding as a mechanism in the analytical interference by CAs in laboratory blood tests [2–4, 6–8, 11–13, 15].

Analytical interference by GdCAs in iron assays has been reported with different agents and in different assays, in which the observed lack of analytical interference by macrocyclic agents on iron determination is supported by the literature [5, 12, 14, 15]. Recent reports have linked the observed negative interference by linear GdCAs to dissociation of the gadolinium complex, which is caused by transmetallation between the ferric ion and gadolinium [15, 42], a mechanism also attributed to the negative interference caused by GdCAs in zinc assays [11, 16]. In this study, only the ionic linear GdCA, gadoxetate disodium, exhibited analytical interference in the zinc assay. Proctor et al. [12] reported analytical interference in zinc assays by the nonionic macrocyclic GdCA, gadoteridol, and by linear agents. No effect was observed in this study with gadobutrol, which is also a nonionic macrocyclic agent. Furthermore, GdCA injections have been linked to zincuria [16], and future studies should focus on resolving whether it is a “factual” in vivo or an “artefactual” in vitro effect.

Analytical interference in the magnesium assay was solely exhibited by the ICA iomeprol, and this is an unreported effect. The lack of interference by the GdCAs is supported by others, as the analytical interference by GdCAs on the determination of magnesium has been credited to the nonionic linear agents [12].

The lower sodium assay results may also be explained by relatively more sodium in the control solution. Sodium was present in both the CA and the physiological saline that was added to adjust the volume. However, the various CA may have less than physiological sodium concentrations. The observed changes in sodium may, therefore, be true and not due to analytical interference.

Studies indicate a lack of awareness in daily clinical work regarding analytical interference by CAs [11, 13]. Spurious results from laboratory assays may cause unnecessary treatment, diagnostic confusion, unnecessary distress to the patient, and increased use of healthcare resources [43]. For these reasons, manufacturers of diagnostic tests should examine for CA interference and document their results. The European Society of Urogenital Radiology Guidelines on Contrast Media recommend that biochemical analyses are avoided on blood and urine collected in the 24 hours after CA injection. The present findings indicate that this recommendation could be reduced to 4 hours, in which 75% of the injected CA has been eliminated in patients with normal renal function [28]. However, it is preferable if the blood and/or urine samples are collected before the CA is administered.

The limitations in this study were as follows: (1) no experiments were performed to understand the underlying mechanism, thus making it difficult to distinguish “factual” in vivo effects from “artefactual” in vitro effects. (2) Due to the varying workload of patient samples in the laboratory, the incubation time could vary between 2 and 4 h. The observed significant effects from incubation time indicate that a reduction in the variation of incubation time would improve both the generated results and the reproducibility. (3) In addition to the calcium assays, this study did not include multiple assays on single analytes and precautions should be taken when comparing the results with those achieved by a different method. (4) The current data do not disclose whether the change in sodium was due to addition, dilution, interference, or a combination thereof. Further experiments taking the sodium concentration of CA into account are required to clarify this question.

In conclusion, some CAs cause analytical interference. Attention should be given to the analyte-CA combinations listed in Table 2 when assessing laboratory blood test results obtained after CA administration. Further research in vivo on the mechanism of action is needed to distinguish between factual and artefactual effects.

## Figures and Tables

**Table 1:** Analysers, biochemical assays, and test tubes (Vacuette Greiner Bio-One).

Analyser	Analyte	Method (Reagents)	Test tubes (Vacuette)
Vitros 5.1 FS Chemistry System, Ortho-Clinical Diagnostics, Johnson & Johnson, Birkerød, Denmark	Alanine amino transferase	Enzymatic, multiple-point rate (pyridoxal-5-phosphate)	LH Lithium Heparin Sep
	Albumin^*∗*^	Direct colorimetry (bromocresol green dye)	LH Lithium Heparin Sep
	Alkaline phosphatase	Multiple-point rate (p-nitrophenyl phosphate)	LH Lithium Heparin Sep
	Aspartate amino transferase	Enzymatic, multiple-point rate (pyridoxal-5-phosphate)	LH Lithium Heparin Sep
	Bicarbonate^*∗*^	Enzymatic, end-point (malate dehydrogenase)	LH Lithium Heparin Sep
	Bilirubin, total^*∗*^	Colorimetry (dyphylline, diazonium salt)	LH Lithium Heparin Sep
	Calcium^*∗*^	Direct colorimetry (arsenazo III dye)	LH Lithium Heparin Sep
	Creatinine	Enzymatic, two-point rate (isotope dilution mass spectrometry (IDMS) standardized)	LH Lithium Heparin Sep
	Glucose	Enzymatic, colorimetry (glucose oxidase method)	FC Mix
	Iron^*∗*^	Two-point rate (3-pyridine sulphonamide dye)	LH Lithium Heparin Sep
	Lactate dehydrogenase^*∗*^	Enzymatic, multiple-point rate (sodium pyruvate)	LH Lithium Heparin Sep
	Magnesium^*∗*^	Colorimetry (formazan dye)	LH Lithium Heparin Sep
	Phosphorus	Colorimetry (phosphomolybdate reduction)	LH Lithium Heparin Sep
	Potassium^*∗*^	Direct potentiometry (valinomycin)	LH Lithium Heparin Sep
	Sodium^*∗*^	Direct potentiometry (methyl monensin)	LH Lithium Heparin Sep
	Urea	Colorimetry (urease, ammonia indicator)	LH Lithium Heparin Sep

Konelab™ PRIME 60i Clinical Chemistry Analyzer, Thermo Fischer Scientific Inc., ILS Laboratories Scandinavia ApS, Allerød, Denmark	C-reactive protein	Turbidimetry, measurement of antigen-antibody precipitation (polyethylene glycol)	Z Serum Sep Clot Activator
	Ionized calcium^*∗*^	Direct potentiometry (ion selective electrode)	Z Serum Sep Clot Activator
	Zinc^*∗*^	Direct colorimetry (dye 5-Br-PAPS)	Z Serum Clot Activator

ACL TOP 700 CTS, Instrumentation Laboratories, Lexington, MA, USA.	Coagulation factors II + VII + X^*∗*^	Turbidimetry (Owren's prothrombin time (PT) reagents)	9NC Coagulation Sodium Citrate

ADVIA 2120i System with Autoslide, Siemens Healthcare Diagnostics, Ballerup, Denmark	Basophilocytes	The haematology assays included flow cytometry and leucocyte differential counting using a peroxidase staining methodology. Hemoglobin, photometric; reagents, potassium cyanide and dimethyllaurylamine oxide	K3E K3EDTA
	Eosinophilocytes		
	Haemoglobin		
	Large unstained cells		
	Leukocytes		
	Lymphocytes		
	Monocytes		
	Neutrophilocytes		
	Thrombocytes		

^*∗*^Analytes reanalysed with serial dilutions of the contrast agent (concentrations of 6 and 3 mg iodine/mL and 0.25 and 0.125 mM gadolinium).

**Table 2:** Assays exhibiting clinically relevant interference by a contrast agent.

	Control	Iodixanol (Visipaque)			Iomeprol (Iomeron)			Gadoxetate disodium (Primovist)		
	Median (IQR)	% change	95% CI (%)	*p* value	% change	95% CI (%)	*p* value	% change	95% CI (%)	*p* value
Albumin, g/L	36 (4)	+1.5	0.5 to 2.5	0.0062						
Bicarbonate, mmol/L	22 (3)	−3.4	−5.9 to −1.0	0.011	−3.9	−6.3 to −1.4	0.0057			
Calcium, mmol/L	2.36 (0.06)	+3.3	2.6 to 4.0	<0.0001	+2.2	1.4 to 3.0	<0.0001			
Ionized calcium, mmol/L	1.18 (0.035)	+1.9	0.7 to 3.2	0.0086						
Iron, *µ*mol/L	15 (3)	+14.3	10.7 to 17.9	<0.0001				−24.6	−28.7 to −20.5	<0.0001
LDH, U/L	191 (32)	−4.8	−6.2 to −3.5	<0.0001						
Magnesium, mmol/L	0.82 (0.03)				−2.7	−3.7 to −1.7	0.0001			
Potassium, mmol/L	4 (0.2)	+3.2	1.9 to 4.5	0.0003	+2.3	1.1 to 3.4	0.0012			
Sodium, mmol/L	141 (3)	−1.1	−1.5 to −0,7	<0.0001	−1.5	−2.0 to −1.1	<0.0001	+0.5	0.1 to 1.0	0.032
Zinc, *µ*mol/L	10 (2)	−5.1	−8.2 to −2.0	0.0034				−32.6	−36.5 to −28.8	<0.0001

IQR, interquartile range; CI, confidence interval; % change, mean percentage change; LDH, lactate dehydrogenase.
